# The Impact of Executive Function on Emotion Recognition and Emotion Experience in Patients with Schizophrenia

**DOI:** 10.4306/pi.2009.6.3.156

**Published:** 2009-06-23

**Authors:** Seung Jae Lee, Hae-Kook Lee, Yong-Sil Kweon, Chung Tai Lee, Kyoung-Uk Lee

**Affiliations:** 1 Department of Psychiatry, Kyungpook National University College of Medicine, Daegu, Korea.; 2 Department of Psychiatry, Uijeongbu St. Mary's Hospital, The Catholic University of Korea College of Medicine, Uijeongbu, Korea.

**Keywords:** Executive function, Emotion recognition, Iowa Gambling Task, Schizophrenia

## Abstract

**Objective:**

This study investigated the impact of executive function on the performance of two different affective tasks, the Facial Affect Identification Task (FAIT) and the Iowa Gambling Task (IGT), in patients with schizophrenia.

**Methods:**

Thirty-nine patients with schizophrenia and 33 healthy controls completed the FAIT and the IGT, followed by the Wisconsin Card Sorting Test (WCST) and the intelligence quotient (IQ) test. In addition to correlation analysis, regression analysis was used to determine the extent to which the performance of the WCST, in particular, perseverative error (PE), accounted for the variation in both the FAIT and the IGT.

**Results:**

Relative to normal controls, patients with schizophrenia showed significant impairments in the IGT, the FAIT and the WCST even after controlling for IQ. While normal controls did not show any relationships between the WCST and two affective tasks, patients with schizophrenia showed that variables in the WCST correlated not only with the FAIT total correct score (r=-0.503, p=0.001 for PE) but also with the IGT net score (r=0.385, p=0.016 for PE). The PE score was a better predictor of the performance on the FAIT (R^2^=0.25) than that of the performance on the IGT (R^2^=0.15).

**Conclusion:**

Our findings imply that deficits in executive function in schizophrenia can affect performance on facial emotion recognition task more than performance on task based on emotion experience, that is, the feedback from the body. Therefore, more consideration is needed of the impact of executive function when interpreting the result of "conventional" facial affect recognition tests as opposed to interpreting the IGT.

## Introduction

Recent advances in neuropsychological assessment and neuroimaging techniques have established cognitive impairment as one important component of the pathophysiology of schizophrenia. In general, patients with schizophrenia display deficits when tested on aspects of higher cognitive functions, such as sustained attention, executive function, working memory, language skills, explicit learning and memory, and perceptual motor processing.^1^,^2^ Goldman-Rakic's work highlighted working memory deficit as a core cognitive impairment, leading to avolition, behavioral disorganization, as well as deficits in conceptual thinking, and memory formation.^3^,^4^ The Wisconsin Card Sorting Test (WCST), among many neurocognitive tasks, has been widely used to explore executive functions in patients with schizophrenia.

For recent two decades, social or affective domains has received growing attention in the field of schizophrenia study, resulting in the emergence of novel neurocognitive tasks to tap these domains including decision-making, facial perception, theory-of-mind skills, and motivation.^5^ However, deficits in cognitive functioning, specifically in executive functions, have confounded the interpretation of the results of these affective tests in patients with schizophrenia. In the face of problems of interpretation, to our knowledge, no study has investigated the impact of the contribution of executive function to perform across variable affective tasks within the same subjects.

Emotion experience emerges from the integration of sensations from the external world with information from the body.^6^ Accordingly, Damasio^7^ proposed that emotion experience arises via a "body loop" or "as-if-loop". In the body loop mechanism, an appropriate somatic state is re-enacted, and signals from the body are relayed back to brain. This mechanism is rather innate and implicit. After emotions are learned, the brain learns to create a fainter image of an emotional body state, without having to re-enact it in the body through the as-if-loop. For example, the facial emotion recognition test requires the subject to consciously match different emotional faces and their relevant emotional words and would engage the "as-if-loop". On the other hand, the Iowa Gambling Task (IGT) would mainly depend on signals from the body proper via a "body-loop" since the decision is made under an uncertain situation.^8^ Therefore, the impact of cognitive function may be dependent on levels or mechanisms of the emotion experience that is probed by the given task.

The IGT, an experimental paradigm developed by Bechara et al.,^9^ is thought to rely on an emotionally mediated "feeling" or "hunch" about which decks are good or bad, in the absence of explicit awareness of the way that the decks pay out.^10^ The relationship between the performance of the IGT and the WCST is still controversial. Although Shurman et al.^11^ reported a significant relationship between the WCST and the choice of a specific deck, most of the studies reported no correlation.^12^-^14^ The rest did not address this issue even though they tested both tasks.^15^-^17^

On the other hand, the facial affect recognition task examines the ability to recognize others' facial expression of emotions. Impaired recognition of facial affect in schizophrenia has been documented extensively.^18^,^19^ With respect to the impact of working memory on the performance of the facial affect recognition task, the facial affect recognition task showed a more consistent, positive correlation with the WCST than did the IGT. For example, Kohler et al.^20^ reported that better emotion recognition performance was correlated with better performance on the WCST. Bryson et al.^21^ found associations between the emotion recognition task score and WCST variables such as categories completed and perseverative errors. Sachs et al.^22^ also reported happy facial emotion recognition correlated with the WCST.

To show the different effects of executive function on different affective tasks at the same time, we chose the IGT and the Facial Affect Identification Tesk (FAIT) because these are among the most frequently applied tasks to measure affective domain and may engage different mechanisms of emotion experience. We hypothesized that the impact of the executive function would be less on the performance of the IGT than on the performance of facial emotion recognition. In this study, we investigated not only the presence of correlation but also the extent to which performance of two different affective tasks were explained by executive function.

## Methods

### Subjects

Thirty-nine stable inpatients with schizophrenia and 33 healthy control subjects recruited from Uijeongbu St. Mary's Hospital, the Catholic University of Korea and Bugok National Mental Hospital and Institutional Review Board were approved for this study. After receiving a complete description of the study, written informed consent was obtained from all participants.

Patients who were about to discharge after remission or who were participating in open ward-based rehabilitation programs were enrolled. The diagnosis of schizophrenia was established by one experienced psychiatrists (S.L.) using the Structured Clinical Interview for DSM-IV (SCID).^23^ Patients having an intelligence quotient (IQ) below 80, with a history of substance use disorder, or with any neurologic and medical disorders known to influence cognitive functioning were excluded.

Regarding antipsychotic medications, 33 patients were taking stable dosages of atypical antipsychotics (risperidone, olanzapine or clozapine), 5 were taking stable dosages of typical antipsychotics (haloperidol or fluphenazine), while 1 was taking both atypical and typical antipsychotics, at therapeutic dosages.

Healthy participants were recruited from staff members in two hospitals as mentioned above. Subjects were screened for psychotic, mood and substance use disorders as well as for history of head injury with significant loss of consciousness (greater than 5 min) or neurological disorder.

Table 1 presents demographic information for both groups and symptom characteristics of the schizophrenia patients. Two groups did not significantly differ in terms of sex and age but scores of IQ in patients were significantly lower than those of normal controls. Symptom assessment at the time of testing revealed low levels of positive and negative psychotic symptoms and general psychopathology in patients.

### Procedures

All participants completed the FAIT and the IGT, followed by the IQ test and the WCST. Two trained psychologists from both hospitals administered the tests. Cognitive tasks were taken directly from the literature and used as described in the original work. Clinical symptom assessments using the Positive and Negative Syndrome Scale (PANSS)^24^ were also conducted for the patient group prior to these tests on the same day. One experienced psychiatrist (S.L.) performed the symptom severity rating.

### Facial Affect Identification Test

The FAIT is a computerized test which uses ChaeLee Korean facial expressions of emotion including happiness, sadness, fear, anger, disgust, surprise and neutral as stimuli. Methods of development and its validation were reported elsewhere.^25^ Briefly, three professional actors and 3 actresses (6 persons in total) participated in the development of the test. They were asked to display each of the following facial expressions in turn: happiness, sadness, fear, anger, disgust and surprise. In order to validate the detection ability of the intended emotion, hundreds of photographs were presented to five healthy raters; 44 photographs were finally chosen on the basis of the consistency of judgments. Following that, the validity of photographs was tested by one hundred persons.

**FIGURE 1:**
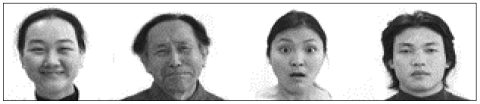
Examples of ChaeLee Korean facial expressions of emotion in the Facial Affect Recognition Test (FAIT): happy, sad, surprise, and neutral facial expressions in order. Note that images in the real test are in full color.

Before the test started, subjects were given an explanation regarding each emotion and furthermore had two practice sessions. Then, subjects were asked to choose one of six emotions and neutral emotion while viewing the images. A total of 44 facial images displaying happy, sad, fear, anger, surprise, disgust, or neutral expressions were then presented: seven faces for surprise and disgust, and 6 faces for the other emotions and the neutral expression (Figure 1). Hence, the maximum score is either 6 or 7 according to the numbers of facial images displayed for each emotion. The order of stimuli was randomized. Choices were displayed on the screen along with the stimuli, and subjects responded by pressing the touch screen.

### Iowa Gambling Task

The IGT^9^,^26^ was designed to evaluate the ability to postpone immediate reward for a longer-term successful outcome, and test decision-making ability based on signals from the body proper since the decision is made under uncertain circumstances. The subjects are instructed that the goal of this game is to win as much money as possible by selecting one card at a time from four decks, namely A, B, C, and D until 100 cards are chosen. However, the subjects do not know when the game will end and play this card game under conditions of limited knowledge regarding both reward and penalty. A and B decks have frequent high gains but also occasional substantial losses, so that sustained playing with these decks leads to overall financial loss. C and D decks have more modest payouts but lead to only small and infrequent loses, so sustained choices from these decks lead to overall moderate gain. A net score is then obtained by subtracting the total number of disadvantageous decks (A and B decks) from the advantageous decks (C and D decks) for all 100 cards.

### Other neuropsychological tests

1) The WCST^27^ assesses the ability to solve problems in response to changing stimuli, the ability to shift and maintain set, and utilize feedback. In this task, participants are required to sort cards according to several different dimensions (color, form, number); the sorting principle must be deduced from verbal feedback provided by the computer. Once a particular response mode is established (i.e., 10 consecutive correct responses), a new sorting principle (concept) is instituted without warning and must be deduced by the participant. Measures of performance included the numbers of categories completed, total and perseverative errors. 2) Vocabulary-Block Design short form is an abbreviated form of the Wechsler Adult Intelligence Scale-Revised (WAIS-R), which calculates an estimated IQ using normative tables.^28^

### Data analysis

The chi-square test (gender) and the t-test (age, education, IQ) were used to compare demographic characteristics between schizophrenia and normal control groups. For the analysis of between-group differences on the FAIT, the IGT, and the WCST was used with IQ as a covariate. Pearson's correlations within each group were performed to find the relation between IQ, variables on the FAIT, the IGT and the WCST. PANSS scores were additionally included in the correlation analysis of schizophrenia group in order to establish relationships between psychiatric symptoms and performance on neuropsychological tests.

Hierarchical regression analysis was used to determine to what extent the performance of the WCST, in particular perseverative error can account for the variation in both the FAIT and the IGT in step 1. The positive score of the PANSS was entered additionally in step 2 because this variable was significantly correlated with the FAIT in the correlation analysis.

Data were analyzed using Statistical Package for Social Science (SPSS) for Windows, version 11.0.1 (SPSS Inc; Chicago, IL, USA). The significance level was established at 0.05.

## Results

### Performances on Facial Affect Identification Task, Iowa Gambling Task, and Wisconsin Card Sorting Test

**FIGURE 2:**
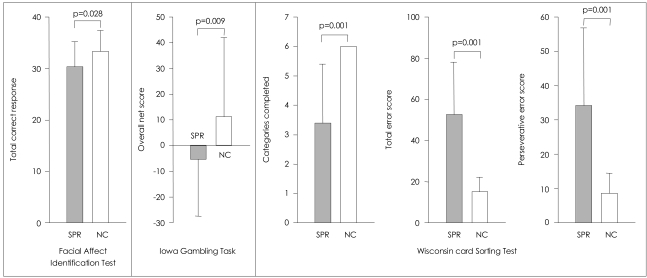
The performances on the Facial Affect Identification Test, the Iowa Gambling Task, and the Wisconsin Card Sorting Test in patients with schizophrenia (SPR) and normal controls (NC).

The performances on the FAIT, the IGT, and the WCST are presented in Figure 2. On the FAIT, the ANCOVA with IQ as a covariate revealed that patients with schizophrenia correctly identified the emotion of overall facial stimuli less often than did controls [F(1,69)=5.04, p=0.028].

On the IGT, patients with schizophrenia had a lower mean overall net score (advantageous minus disadvantageous deck selection) than control subjects [F(1,69)=7.20, p=0.009]. Such a result indicates that healthy participants chose more cards from the advantageous decks (C, D) rather than from the disadvantageous decks (A, B), but schizophrenic patients did not.

The patients, when compared to healthy subjects, exhibited significantly poorer performance on all of the three variables on the WCST [F(1,69)=20.20, p<0.001 for perseverative errors; F(1,69)=42.06, p<0.001 for total errors; F(1,69)=30.25, p<0.001 for categories completed].

### Relationship between the Facial Affect Identification Task and other psychological tests within each group

Higher total scores on the FAIT correlated with better performance on the WCST in patients with schizophrenia [r=-0.503, p=0.001 for perseverative errors; r=-0.508, p=0.001 for total errors; r=0.535, p<0.001 for categories completed]. The correct responses on the FAIT were also negatively correlated with positive scores of PANSS [r=-0.334, p=0.038]. However, normal controls did not show any correlations between the WCST and the FAIT (Table 2 and 3).

### Relationship between the Iowa Gambling Task and other psychological tests within each group

In patients with schizophrenia, contrary to the relationship between the FAIT and the WCST, higher net scores on the IGT correlated with poorer performance on the WCST [r=0.385, p=0.016 for perseverative errors; r= 0.365, p=.023 for total errors]. Unlike the FAIT, no correlations of the IGT with any PANSS scores were found. Normal controls did not show any correlations with the IGT (Table 2 and 3).

### Effects of perseverative error on the Wisconsin Card Sorting Test on the performance of the Facial Affect Identification Task and the Iowa Gambling Task

**FIGURE 3:**
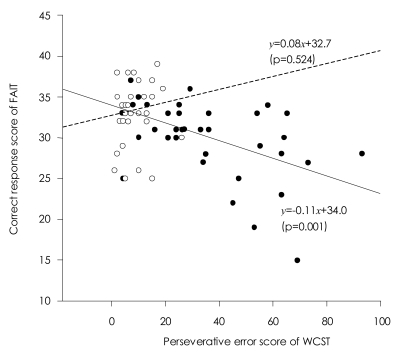
Scatterplot showing the relationship between the Facial Affect Identification Tesk (FAIT) and the Wisconsin Card Sorting Test (WCST) in patients with schizophenia (black circle with solid regression line) and normal controls (blank circle with dotted regression line).

**FIGURE 4:**
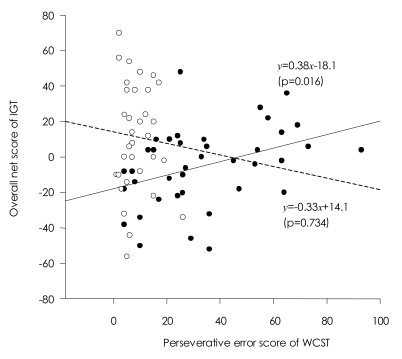
Scatterplot showing the relationship between the Iowa Gambling Task (IGT) and the Wisconsin Card Sorting Test (WCST) in patients with schizophenia (black circle with solid regression line) and normal controls (blank circle with dotted regression line).

Regression analysis indicated that perseverative error was a significant predictor of the performance on the FAIT and the IGT, yet in opposite directions. Perseverative error score accounted for 25% of the total variance in the performance on the FAIT. When including positive scale of PANSS, two variables accounted for 43% of the performance on the FAIT. In regard to the IGT, perseverative error score only accounted for 15% of the total variance in the performance on the IGT (Table 4 and 5). Scatterplots are presented in Figures 3 and 4.

## Discussion

We investigated the impact of executive function on the performances of two different affective tasks, the FAIT and the IGT, in patients with schizophrenia. The main findings were that:

1) Relative to normal controls, patients with schizophrenia showed significant impairments in the IGT, the FAIT and the WCST even after controlling for IQ. 2) While normal controls did not show any relationships between the WCST and two affective tasks, in patients with schizophrenia variables in the WCST correlated not only with the FAIT but also with the IGT. 3) Perseverative error was a better predictor of the performance on the FAIT than performance on the IGT.

Although the effects of executive function on several affective tasks in patients with schizophrenia have not been well established, facial affect recognition tasks generally have shown consistent and positive correlation with the WCST. For example, Bryson et al.^21^ demonstrated that subjects with more severe impairment of affect recognition displayed more perseverative errors (r=0.33, p<0.01), and had fewer categories completed (r=0.33, p<0.01) on the WCST.^23^ In line with previous observations,^20^-^22^,^29^,^30^ we replicated the finding that the FAIT showed significant correlations with all three variables of the WCST in patients with schizophrenia. These findings suggest that subjects who are prone to switch cognitive sets and maintain the task on the WCST are better able to select the appropriate emotional label and change that label on each subsequent facial recognition trial. One possible explanation may be based on the design of the task. Since most affect recognition tasks including ours employ a design requiring the subject to choose the proper term for the given facial affect picture at every trial, processing online information of newly presented pictures and discarding the unnecessary information from previous pictures occur repeatedly throughout the whole trials. According to the neural system for face perception proposed by Haxby and colleagues,^31^ the prefrontal cortex is not a core neural region for face perception. Instead, prefrontal regions may be activated when subjects are engaged in a cognitive task requiring explicit identification of the emotion.^32^

More importantly, we found that only a single variable, the perseverative error score, accounted for 25% of the variance in the performance on the FAIT in patients with schizophrenia. Our results suggest that the impact of executive function should be considered in interpreting the result of "conventional" face affect recognition tests and that a new paradigm to probe facial affect recognition with less interference of working memory needs to be developed.

With respect to the relationship between the IGT and the WCST, better performance on the IGT paradoxically correlated with poorer performance on the WCST in patients with schizophrenia. One possible explanation for this remarkable finding might be that those with better performance on the WCST rely on both emotional and cognitive sources of evidence, while those with poorer performance on the WCST rely unduly on emotion-based sources.^33^ Thus, the IGT is designed to tap only one of the theses sources of information (emotion-based knowledge) so that a substantial benefit is added to for poorer WCST performers over a period of time.^34^ However, this is an oversimplified explanation when considering the complexity of decision-making processes. In real-world problems solving situations, over-reliance on emotion-based resources is likely to lead to the generation and maintenance of false beliefs.^33^,^34^ In effect, most of previous papers directly correlating indices from the IGT with WCST scores failed to find any correlations,^12^-^14^ supporting the proposal of Bechara et al.^9^,^35^ that performance on the IGT is relatively independent of cognitive functions such as executive function believed to be associated with the dorsolateral prefrontal cortex. This notion is also supported by our result that unlike the FAIT, the perseverative error score only accounted for 15% of the total variance in the performance on the IGT.

Our study has several limitations. The patient group included the heterogenous subtypes of schizophrenia. Indeed, Bark et al.^16^ revealed that catatonic schizophrenic patients showed deficits in the IGT while paranoid patients did not. Thus, this heterogeneity might have affected our results. Cognitive deficits shown in the patients may arise from the use of antipsychotics and anticholinergic drugs. The use of different antipsychotics may also lead different results. Beninger et al.^12^ found that 18 patients on atypical antipsychotics demonstrated impairments similar to patients with orbitofrontal cortex lesions whereas 18 patients on typical antipsychotics did not significantly differ from controls on a decision-making task, stating that the existence of dysfunction on decision-making is related to the kind of antipsychotic medication.

In conclusion, we showed that perseverative error score accounted for 25% of the total variance in the performance on the FAIT rather than for 15% in the performance on the IGT. Our findings imply that deficit in executive function in schizophrenia can affect performance on a facial emotion recognition task more than on a task based on emotion experience, that is, the feedback from the body. Therefore, careful consideration of the impact of executive function is necessary when interpreting the result of "conventional" face affect recognition test, more so than when interpreting the IGT.

## Figures and Tables

**TABLE 1:**
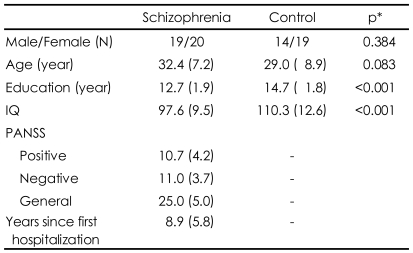
Demographic and clinical information for schizophrenia and control groups

Standard deviations appear in parentheses. ^*^χ^2^ for categorical variables, t-test for continuous variables. PANSS: the positive and negative syndrome scale, IQ: intelligence quotient

**TABLE 2:**
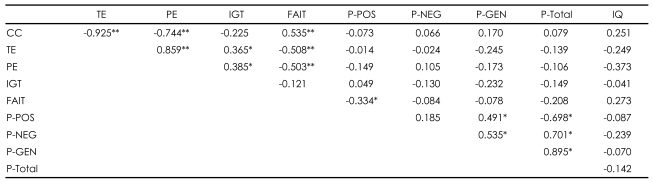
Relationships between psychological tests within schizophrenia group (N=39)

^*^p<0.05, ^**^p<0.01. CC: categories completed, TE: total error, PE: perseverative error, IGT: total net score on the Iowa Gambling Task, FAIT: total correct number on Facial Affect Identification Test, P-POS: PANSS positive score, P-NEG: PANSS negative score, P-GEN: PANSS general pathology score, P-total: PANSS total score, IQ: intelligence quotient, PANSS: Positive and Negative Syndrome Scale

**TABLE 3:**
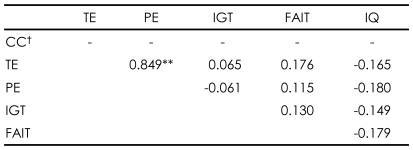
Relationships between psychological tests within normal control group (N=33)

^**^p<0.01, ^†^Cannot be computed because the mean of numbers of categories completed is constant. CC: categories completed, TE: total error, PE: perseverative error, IGT: total net score on the Iowa Gambling Task, FAIT: total correct number on Facial Affect Identification Test, IQ: intelligence quotient

**TABLE 4:**
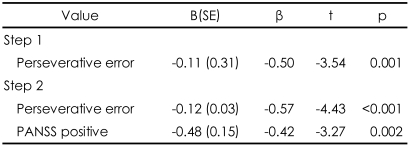
Regression analysis of the performance of the Facial Affect Identification Test in relation to perseverative error of the Wisconsin Card Sorting Test in schizophrenia group

R^2^=0.25 for step 1 (p=0.001); R^2^=.43 for step 2 (p<0.001). B (SE): unstandardized coefficients (standard error), β: standardized coefficients

**TABLE 5:**
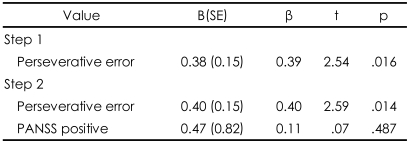
Regression analysis of the performance of the Iowa Gambling Task in relation to perseverative error of the Wisconsin Card Sorting Test in schizophrenia group

R^2^=0.15 for step 1 (p=0.016); R^2^=0.16 for step 2 (p<0.044). B (SE): unstandardized coefficients (standard error), β: standardized coefficients
